# Targeted delivery and ROS-responsive release of Resolvin D1 by platelet chimeric liposome ameliorates myocardial ischemia–reperfusion injury

**DOI:** 10.1186/s12951-022-01652-x

**Published:** 2022-10-20

**Authors:** Xueyi Weng, Haipeng Tan, Zheyong Huang, Jing Chen, Ning Zhang, Qiaozi Wang, Qiyu Li, Jinfeng Gao, Dili Sun, Wusiman Yakufu, Zhengmin Wang, Weiyan Li, Guangrui Zhu, Zhiqing Pang, Yanan Song, Juying Qian, Junbo Ge

**Affiliations:** 1 Department of Cardiology, Zhongshan Hospital, Shanghai Institute of Cardiovascular Diseases , Fudan University, 180 Feng Lin Road, Shanghai, 200032 China; 2 National Clinical Research Center for Interventional Medicine & Shanghai Clinical Research Center for Interventional Medicine, 180 Feng Lin Road, Shanghai, 200032 China; 3 School of Pharmacy, Key Laboratory of Smart Drug Delivery, Ministry of Education, Fudan University, 826 Zhangheng Road, Shanghai, 201203 China; 4 Institute of Biomedical Science, Fudan University, 180 Feng Lin Road, Shanghai, 200032 China

**Keywords:** Biomimetic, ROS responsive, Macrophages, Monocytes, Myocardial ischemia–reperfusion injury, Platelets

## Abstract

Resolvin D1 (RvD1) has been shown to provide effective protection against ischemia–reperfusion injury in multiple vital organs such as the heart, brain, kidney. However, the clinical translational potential of systemic administration of RvD1 in the treatment of ischemia–reperfusion injury is greatly limited due to biological instability and lack of targeting ability. Combining the natural inflammatory response and reactive oxygen species (ROS) overproduction after reperfusion injury, we developed a platelet-bionic, ROS-responsive RvD1 delivery platform. The resulting formulation enables targeted delivery of RvD1 to the injury site by hijacking circulating chemotactic monocytes, while achieving locally controlled release. In a mouse model of myocardial ischemia repefusuin (MI/R) injury, intravenous injection of our formula resulted in the enrichment of RvD1 in the injured area, which in turn promotes clearance of dead cells, production of specialized proresolving mediators (SPMs), and angiogenesis during injury repair, effectively improving cardiac function. This delivery system integrates drug bio-protection, targeted delivery and controlled release, which endow it with great clinical translational value.

**Supplementary Information:**

The online version contains supplementary material available at 10.1186/s12951-022-01652-x.

## Introduction

Myocardial infarction (MI), a clinical manifestation of coronary heart disease, is the leading cause of cardiovascular-related deaths [1]. Timely reperfusion therapy can effectively reduce myocardial loss and significantly reduce acute mortality in patients with MI [2]. However, reperfusion concurrently elicits multiple adverse events, so that a significant proportion of surviving patients eventually head to heart failure. These events primarily include metabolic alterations, reactive oxygen species (ROS) overproduction, autophagy dysregulation, and mitochondrial dysfunction, all of which lead to additional cardiomyocyte death and an excessive inflammatory response [3–5]. Efficient removal of dead cells and timely resolution of inflammation are exactly the keys to the restoration of cardiac function after myocardial ischemia–reperfusion (MI/R) injury [6–8].

Resolvin D1 (RvD1), a member of specialized proresolving mediators (SPMs) that has recently been suggested to lead active resolution of acute inflammation, has been shown to promote the clearance of apoptotic and necrotic cells by macrophages [9–12]. Interestingly, RvD1 can also promote the synthesis of other SPMs, creating a positive feedback that promotes inflammation resolution [13, 14]. Furthermore, one recent report revealed that RvD1 can promote the differentiation of macrophages to a pro-angiogenic phenotype, thereby promoting injured tissue repair [15]. All of the above make RvD1 a potential small molecule drug targeting macrophages to promote cardiac repair after MI/R injury. Correspondingly, RvD1 has been reported to provide effective protection for multiple vital organs such as the heart, brain, and kidney during ischemia–reperfusion injury [16–20]. However, the short half-life due to small molecular weight and rapid enzymatic degradation in vivo limits the therapeutic potential of this molecule [10]. In addition, when RvD1 is administered systemically, the limited target retention determines the need for higher drug concentrations to maintain efficacy, which may bring some potential adverse effects. Furthermore, the release of the drug at the proper time and place is also one of the determinants of its effectiveness.

Herein, we aimed to develop an RvD1 delivery strategy that integrates biosafety, piggyback efficiency, targeted delivery and controlled release. Firstly, considering the advantages of lipid-based drug delivery systems such as biodegradability, low toxicity, long circulation time, flexible synthetic methods, content protection, and easy modification, we adopted liposomes as the backbone of the RvD1 delivery platform [21–25]. Secondly, the number of macrophages, as the main effector cells of RvD1, began to increase and influential only after 2 days and later, while the enhanced permeability and retention (EPR) effect was significantly diminished 24 h after MI/R injury [26, 27]. This mismatch makes targeting monocytes/macrophages challenging after MI/R injury. We have recently developed a platelet biomimetic targeting system that can actively target macrophages in damaged hearts by riding circulating monocytes, independent of enhanced permeability and retention (EPR) effects [28]. On this basis, we propose a method of platelet membrane chimerism to modify liposomes to achieve targeted delivery of RvD1 to cardiac macrophages at the injured site. In addition, given the large amount of ROS generated upon MI/R, we modified the liposomes by incorporating ROS-sensitive diselenide bonds into the lipid components to achieve localized ROS-responsive release of RvD1 [29].

**Fig. 1:**
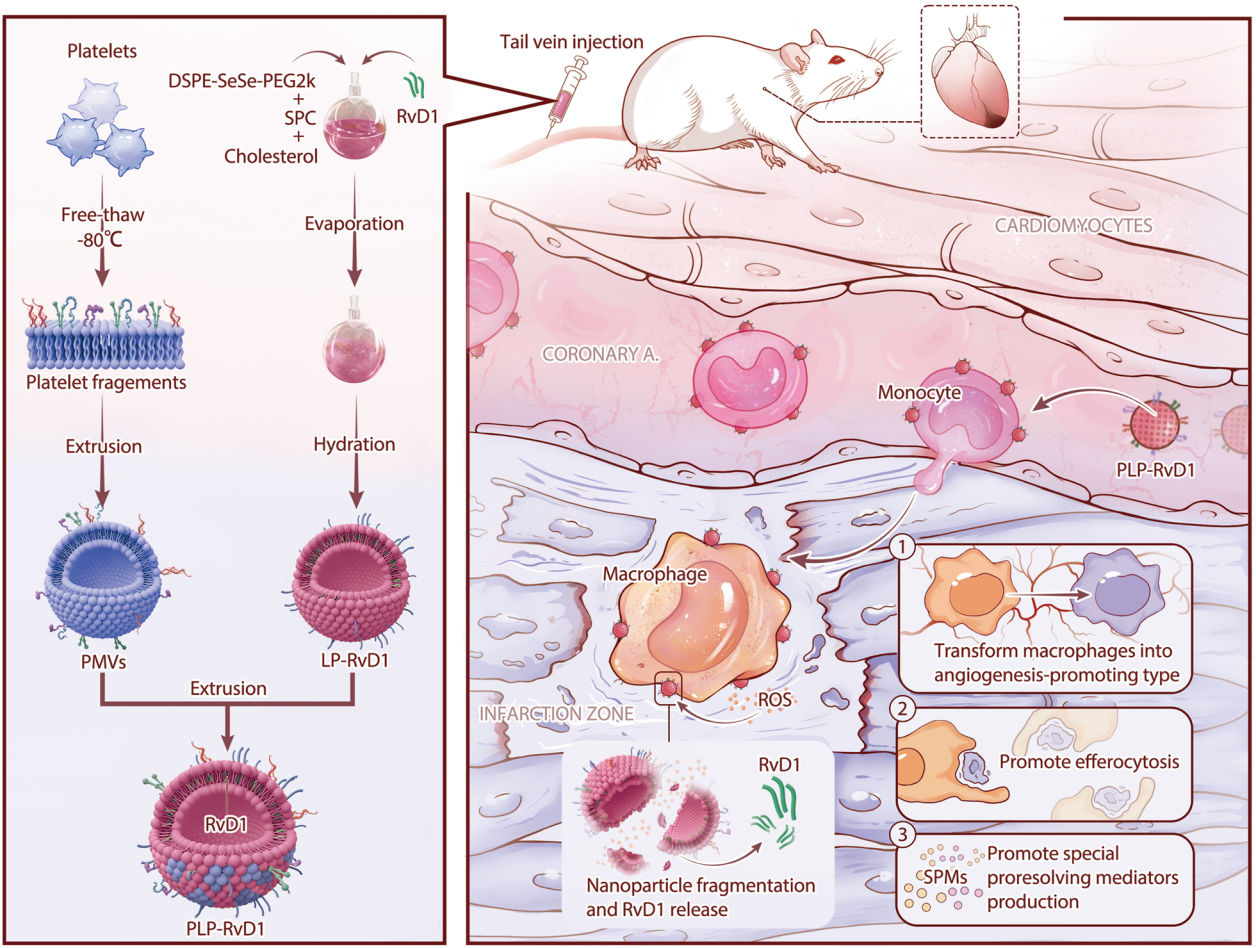
Schematic of PLP-RvD1 fabrication and its targeting treatment for myocardial ischemia–reperfusion injury

Collectively, we designed a platelet-bionic, ROS-responsive RvD1 (PLP-RvD1) delivery platform, which is obtained by chimerization of RvD1-loaded ROS-responsive liposomes and platelet membranes (Fig. 1). The delivery platform inherits the ability of platelets to interact with monocytes so that they can reach the site of cardiac injury by riding chemotactic circulating monocytes after intravenous injection. The large amount of ROS at the injured area disintegrates the delivery platform, enabling rapid release of RvD1. The released RvD1 promotes the clearance of dead cardiomyocytes, SPM production, and angiogenesis, which in turn can effectively improve ventricular remodeling and preserve cardiac function in MI/R-induced mice.

## Materials and methods

### Synthesis and characterization of PLP-RvD1

To fabricate PLP-RVD1, liposomes (LPs) were prepared by the lipid film rehydration process first [30]. Briefly, 9 mg of Soya phosphatidylcholine (SPC) (HY-125853, Med Chem Express), 1.5 mg of 1,2-distearoyl-sn-glycero-3-phosphoeth-anolamine-N-SeSe-(polyethyleneglycol-2000) (DSPE-SeSe-PEG2000) (R-DZP014, Xi’an Ruixi Biological Technology) and 1.5 mg cholesterol (HY-N0322, Med Chem Express) were dissolved in 12 ml of chloroform and dried in a flask by rotary evaporation for 30 min. Then the thin lipid film was hydrated with 12 ml of distilled water at 37 ℃ for 15 min and sonicated at a frequency of 52 kHz and a power of 100 W for 15 min. The lipid suspension was extruded 20 times through 1.0 um, 0.4 um and 0.2 um polycarbonate membrane (Nuclepore Track-Etched Membranes, Whatman) successively to assemble a clear suspension of lipid by using a LiposoFast extruder apparatus (Avestin). The platelets (PLTs) were collected from platelet rich plasma and platelet membrane fractions were derived by an 80℃ repeated freeze–thaw process as described previously [30]. The obtained platelet membrane fractions were successively extruded 20 times through 1.0 um, 0.4 um and 0.2 um polycarbonate membrane to obtain platelet membrane vesicles (PMVs). After centrifugation at 5000 *g* for 15 min, the obtained PMVs were resuspended in phosphate buffer saline (PBS) for further use. To achieve chimerism of PMVs and LPs, PMVs were incubated with LPs at a mass ratio (total protein: lipid) of 1:500 in an ultrasonic bath at room temperature for 20 min and then extruded by the same method.

The obtained PMVs and LPs were hybridized with through an incubation extrusion method.

Fluorescence dye-labeled or RvD1 loaded LPs (LP-RvD1) were prepared by using the same method except that 250 μg of 19-dioctadecyl-3,3,39,39tetramethylindodica-rbocyanine perchlorate (DiD) or 250 μg of RvD1 (R912909, MACKLIN) were added in the initial lipid solution. As a control, non-ROS-responsive liposomes were prepared by using DSPE-PEG2000 (HY-140741, Med Chem Express) instead of DSPE-SeSe-PEG2000.

The RvD1 loading efficiency was determined by high performance liquid chromatography (HPLC). Briefly, samples containing RvD1 were injected in the RP-C18 column and eluted using a binary gradient of methanol and water (total flow rate of 0.40 ml/min). The retention time of RvD1 was 46 min. In the release study, we validated the RvD1 release properties of PLP-RvD1 in PBS, 20% serum, and 20% serum + pathological concentrations (100 uM) of H_2_O_2_. Non-ROS-responsive PLP-RvD1 without diselenide bonds in 20% serum + H_2_O_2_ served as a control. Prepared PLP-RvD1 were equally divided in several tubes, and at different time points, the samples were centrifuged at 100,000 *g* to obtain the pellets. Then ethanol was used to extract RvD1 from the pellets and the extracted RvD1 mimics were analyzed by HPLC.

The morphology and size of nanoparticles were observed under a transmission electron microscope (TEM, Tecnai G2 Spirits Twin, FEI) after negative staining with 2% sodium phosphotungstate solution. The size distribution and zeta potential were determined by a dynamic light scattering (DLS) detector (Zetasizer-ZS90, Malvern Instruments).

To analyze the protein composition in PLP-RvD1, 20 ug protein samples were loaded on 10% SDS-PAGE gel electrophoresis. The gel was stained by coomassie brilliant blue R-250 and decolored until the bands were visualized clearly. We use western blot to verify typical proteins that mediate the interaction between platelets and macrophages on PLP-RvD1. Briefly, the samples were separated on 10% SDS polyacrylamide gel (BioRad), then transferred to polyvinylidene fluoride (PVDF) membrane, and then detect with specific antibody for GP IIb/IIIa (sc-21783, Santa Cruz), P-selectin (sc-8419, Santa Cruz), GP Ibα (4067-GP-050, R & D Systems), and CD42c (ab96565, Abcam). After stained with horseradish peroxidase (HRP)-conjugated secondary antibodies (Biotech Well) with corresponding species reactivity, blots were imaged using a Bio-Rad Chemi DocTM imaging system.

In view of WGA could selectively bind the N-acetylneuraminic acid residues and N- acetylglucosamine of glycoproteins, fluorescent WGA was used as a probe to verify the right orientation of the membrane proteins on the PLP-RvD1. The fluorescence intensity of each sample was measured using a Multiplate Reader (Molecular Devices).

### Cell culture

Murine bone marrow-derived macrophages (BMDMs) were extracted from 8-week-old male C57BL/6 mice according to previously reported protocol [31].Cells were cultured in Dulbecco’s modified Eagle’s medium (DMEM) supplemented 10% of fetal bovine serum (FBS), 1% penicillin/streptomycin (P/S) and 20 ng/ml recombination murine Macrophage Colony Stimulating Factor (M-CSF, PeproTech). After 7 days, the mature macrophages were obtained and the successful extraction of cells was confirmed by flow cytometry (FCM). Proinflammatory BMDMs activation was achieved by stimulating mature BMDMs with 100 ng/ml lipopolysaccharide (LPS, Sigma) and 20 ng/ml Interferon-Gamma (IFN-γ, PeproTech) for 24 h. Human umbilical vein endothelial cells (HUVECs, ATCC) were cultured using Endothelial Cell Medium (ECM, Sciencell). Mouse atrial myocyte (HL-1, ATCC) were cultured in Dulbecco’s modified Eagle’s medium (DMEM) supplemented 10% of FBS and 1% P/S. Cells were all cultured in a humidified cell culture incubator (ThermoFisher Scientific) at 37 ℃ with 5% carbon dioxide (CO2).

### PLP-RvD1 adherence to macrophages in vitro

To examine the binding ability of PLP-RvD1 to BMDMs in vitro, BMDMs were pro-inflammatory activated by the previously described method. Inflamed BMDMs were incubated with 200 ul PBS, DiD (on liposome) labeled LP-RvD1 or PLP-RvD1, respectively, for 30 min. The interaction between nanoparticles and cells was analyzed by immunofluorescence assay. The cell membrane was stained with cell membrane staining kit (Biotium) to detect the membrane colocalization relationship. DAPI (Beyotime) was used as a nuclei indicator. Fluorescence signals were detected by a confocal laser scanning microscope (CLSM, Olympus).

### Efferocytosis assay in vitro

We used fluorescent tracing to evaluate the effect of PLP-RvD1 on promoting macrophage efferocytosis in vitro. First, after 30 min treatment of BMDMs with 200 ul PBS, LP-RvD1 or PLP-RvD1, a pathological dose (100 uM) of H2O2 was administered to induce responsive release of RvD1, followed by incubation for 6 h. Second, HL-1 cardiomyocytes were induced to undergo apoptosis with 5 uM Staurosporine (HY-15141, Med Chem Express) after labeling with IVISense 680 Fluorescent Cell Labeling Dyes (NEV12000, Perkinelmer). It was then incubated with Calcein AM (ab141420, Abcam) labeled BMDMs, and co-localization was observed under a confocal microscope to assess the efferocytosis of macrophages. We calculated the percentage of cardiomyocyte-associated macrophages. DAPI (Beyotime) was used as a nuclei indicator.

### Angiogenesis assay in vitro

Tube formation and migration assays were used to explore pro-angiogenic effects. To verify the effect of PLP-RvD1 on angiogenesis by influencing macrophages, we adapted transwell system to achieve the coculture of HUVECs and BMDMs. First, after 30 min treatment of BMDMs with 200 ul PBS, LP-RvD1 or PLP-RvD1, a pathological dose (100 uM) of H2O2 was administered to induce responsive release of RvD1, followed by incubation for 6 h. At the same time, HUVECs was subjected to 6 h of hypoxic conditions (1% O2). Then, BMDMs treated in various ways were added to the inner chamber, and HUVECs after hypoxia were added to the basolateral chamber, and co-cultured for 12 h. HUVECs cultured under normoxia were adopted as positive control. After that, the HUVECs of the lower chamber were collected. For capillary formation, 48-well cell culture plates were coated with Growth factor reduced Matrigel (Corning) and incubated for 30 min at 37 ℃ to promote gelation. The obtained HUVECs were detached using 0.25% trypsin and subsequently plated at 5 × 10^4^ cells per well on the Matrigel for 6 h at 37 °C. Capillary tubes were observed using a light microscope (Leica). For cell migration assay, HUVECs were seeded on 6-well culture plates, grown until confluence. After HUVECs undergo the same treatment as described above, a linear gap was created by scratching the surface of the culture plates using a sterile yellow tip. The cells were rinsed three times with PBS to thoroughly remove the detached cells. Scratches were photographed at two time points (0 and 12 h) and relative migration areas were quantified using the following formulation: [(initial cell-free area − cell-free area at 12 h)/initial cell-free area] × 100%. In the above two assays, quantitative analysis was performed based on six random fields of view by using ImageJ software.

### Animals

Male C57 BL/6 mice and male ICR mice were all purchased from Shanghai Jiesijie Laboratory Animal, co. Ltd. All animal experiments were approved by the Ethics Committee of Zhongshan Hospital, Fudan University, Shanghai, China and in compliance with the guidance for the Care and Use of Laboratory Animals published by the National Research Council (U.S.) Institute for Laboratory Animal Research.

### Mice model of MI/R

The mice model of MI/R was established as follows. Briefly, mice were anesthetized with ketamine (100 mg/kg, i.p.) and were positive-pressure ventilated by small animal respirator after tracheal intubation. The exposed heart was prepared by opening left thorax and then the left anterior descending coronary artery was ligated for 60 min with 6–0 silk suture which was then released to allow coronary reperfusion. Successful acute MI/R was confirmed by ST-segment characterized electrocardiogram and left ventricle color alteration.

### Biodistribution and targeting specificity of PLP-RvD1 in vivo

To perform pharmacokinetic and biodistribution study, male ICR mice randomly assigned to 2 groups were intravenously administered 200 μL of DiD (on liposome) labeled LP-RvD1 or PLP-RvD1, respectively. For blood circulating profile, blood samples were collected at preset time points (1 min, 5 min, 15 min, 30 min, 1 h, 3 h, 6 h, 12 h, 24 h and 48 h) via cheek pouch puncture, and the fluorescence intensity of blood samples was determined by a Multiplate Reader (Molecular Devices, USA).

To analyze the distribution of LP-RvD1 and PLP-RvD1 in main organs (including heart, liver, spleen, lung, kidney and brain), MI/R induced C57 BL/6 mice randomly assigned to 3 groups were intravenously administered 200 μl of PBS, DiD (on liposome) labeled LP-RvD1 or PLP-RvD1, respectively (n = 6 per group). At 3 h post administration, major organs were harvested and then ex vivo imaged by the In vivo spectrum imaging system (IVIS) (PerkinElmer, Inc., Waltham, MA). The distribution of nanoparticles in the injured heart was also evaluated by immunofluorescence staining. The injured heart was harvest and embedded in optimal cutting temperature compound (OCT, Sakura Finetek), and then frozen in liquid nitrogen before being cut into 8-um cryosections. For immunofluorescence staining, the obtained tissue sections were co-stained with anti-cardiac troponin T antibody (15,513–1-AP, ProteinTech) to identify the infarct area, and a nuclei indicator, DAPI. Then, sections were visualized under fluorescence microscopy.

To explore the potential mechanism of selective accumulation of PLP-RvD1 in damaged heart, we used flow cytometry to detect the binding of PLP-RvD1 to circulating monocytes. Thirty minutes after the injection, the blood of the mice was stained with specific antibodies, FITC-anti-CD45 (#561,867, eBioscience), PerCP-Cy5.5-anti-CD11b (#45–0112-82, eBioscience) and PE-Cy7-anti-Ly6C (#560,593, BD Pharmingen), then treated with RBC Lysis Buffer (Invitrogen) for 5 min and fluorescence signals were detected by BD FACS Aria III. To further explore the pathway of PLP-RvD1 to reach the myocardial infarction area, we performed a colocalization analysis on the nanomaterials and monocytes/macrophages in the injured area by using immunofluorescence staining. Rabbit anti-CD11b antibodies (ab128797, Abcam) and Alexa Fluor 488-anti-Rabbit secondary antibody (ab150077, Abcam) were used. Fluorescence signals were detected by CLSM and colocalization analysis of the images was performed by ImageJ.

### Efferocytosis assay in vivo

To directly assess the clearance capacity of macrophages against apoptotic cardiomyocytes, we employed a previously established method to quantify efferocytosis in situ [32]. Similar to our ex vivo analyses, we enumerated the percentage of cardiomyocyte-associated macrophages by costaining myocardial sections for cardiomyocyte Desmin (D1033, Sigma) and macrophage F4/80 (ab6640, Abcam). The analysis was performed in a blinded fashion by 2 independent observers.

### Pro-angiogenesis efficiency of PLP-RvD1 in vivo

To determine the angiogenesis in injured heart, we divided MI/R induced mice into 3 groups randomly (n = 6 per group) and were treated with 200 μl PBS, LP-RvD1 or PLP-RvD1, respectively. 7 days after injection, the heart was harvest. The obtained tissue sections were stained for CD31(ab28364, Abcam), a typical mature endothelial marker, and DAPI. Fluorescence signals were detected by CLSM.

### SPMs quantification in vitro and in vivo

The levels of RvD1, RvD2, RvE1, lipoxin B4 in BMDMs medium (in vitro) and cardiac tissue homogenate (in vivo) were detected by ELISA assay. Resolvin D1 EIA Kit (500,380, Cayman), Resolvin D2 ELISA Kit (501,120, Cayman), Resolvin E1 ELISA Kit (TW10239, TW-Reagent) and Lipoxin A4 ELISA Kit (E-EL-0053c, Elabscience) were used according to instruction.

### Cardiac protection efficiency of PLP-RvD1

Cardiac function of MI/R mice models was first evaluated by transthoracic echocardiography (Visual Sonics, Vevo 770) 1 day before and 4 weeks after treatment. Mice were anesthetized with low-dose isoflurane, left ventricular ejection fraction (LVEF) and fraction shortening (LVFS) were calculated in six consecutive cardiac cycles, following two-dimensional targeted M-mode traces at the papillary muscle level. Then, mouse hearts were harvested and cut into 5-μm sections, fibrosis extension and infarct size were visualized and measured by a common histochemical procedure including Masson.

### Biosafety analysis of PLP-RvD1

To test the immunoregulation effects of PLP-RvD1, healthy C57 B/L mice were randomized and treated with 200 μl PLP-RvD1 or PBS (n = 6 per group), respectively. Mice were sacrificed, and serum was obtained at the indicated times. The serum level of inflammatory cytokines TNF-α, IL-1β and general antibodies immunoglobulin G (IgG) and IgM were detected by ELISA (BioLegend, USA). To assess the potential impact of PLP-RvD1 on coagulation function, whole blood anticoagulated with sodium citrate was analyzed by activated partial thromboplastin time (APTT), prothrombin (PT), and fibrinogen levels (Fbg). For organ toxicity, biochemical tests of liver (aspartate amino-transferase, AST; alanine amino-transferase, ALT) and renal function (creatinine, CREA; urea nitrogen, UREA) were performed on the serum of mice after treated with PBS or PLP-RvD1. Meanwhile, histopathological changes in major organs were observed by HE staining.

### Statistical analysis

Unless otherwise specified, the data were presented as mean ± SD. Comparisons between any two groups were made using two-tailed unpaired Student’s t test. Multiple groups were compared by one-way analysis of variance, and then use the Bonferroni test to analyze the statistical difference between any two groups. When p < 0.05, the difference was considered statistically significant. Statistical analysis was performed using graphpad prism 8.0.

## Results and discussion

### Fabrication and characterization of PLP-RvD1

**Fig. 2:**
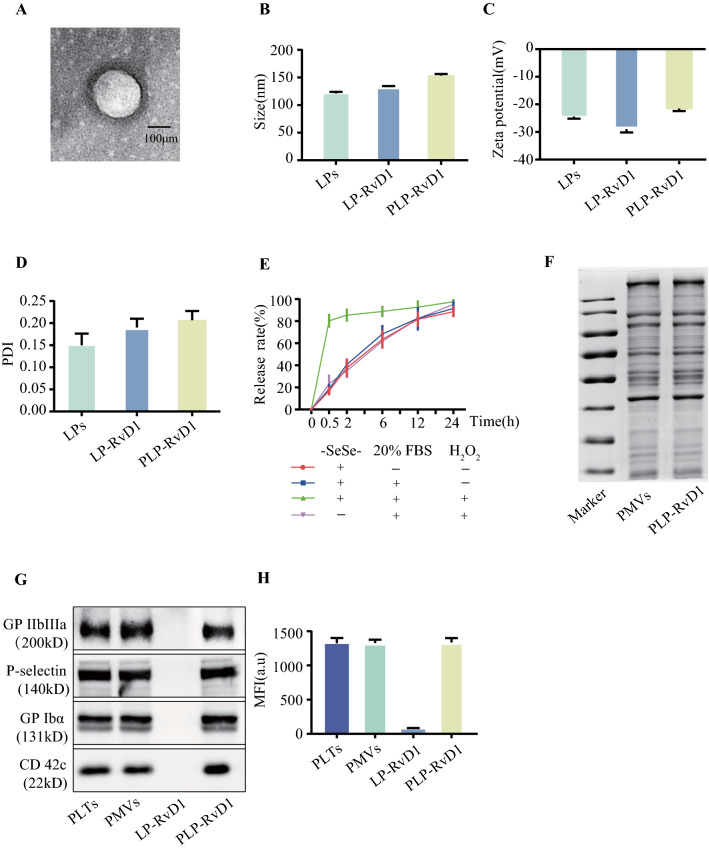
Preparation and Characteristics of PLP-RvD1. **A** TEM of PLP-RvD1. **B** Size of LPs, LP-RvD1 and PLP-RvD1 (n = 3 per group). **C** Zeta potential of LPs, LP-RvD1 and PLP-RvD1 (n = 3 per group). **D** Polymer dispersity index of LPs, LP-RvD1 and PLP-RvD1 (n = 3 per group). **D** Release profiles of RvD1 from nanovesicles (n = 3 per group). **F** Protein content visualization of PMVs and PLP-RvD1 running on SDS-PAGE at equivalent protein concentrations followed by Coomassie staining. **G** Western Blot of five key proteins in PLTs, PMVs, LP-RvD1, and PLP-RvD1 that mediate the interaction between PLTs and macrophages. **H** The glycosylated membrane proteins with correct orientation on PLTs, PMVs, LP-RvD1, and PLP-RvD1 were stained with Texas Red-X-conjugated WGA (n = 3 per group). All data expressed as Mean ± SD

As shown in Fig. 1, LPs were synthesized by the thin-film hydration [33]. RvD1 was loaded in the process of liposome preparation to construct LP-RvD1, and the chimerizaction of platelet membrane vesicles (PMVs) with liposomes was achieved by extrusion. During the entire process of formula construction, the morphology, size and zeta potential of the nanoparticles were dynamically monitored. The ultimate morphology of PLP-RvD1 was spherical under transmission electron microscope (TEM) (Fig. 2A). The size and zeta potential of LPs measured by dynamic laser scattering (DLS) were 120.93 ± 2.99 nm and − 24.40 ± 0.79 mV, respectively. With the loading of RvD1, the size of the nanoplatform increases to 133.187 ± 1.16 nm, and the negativity of potential also increased to − 28.97 ± 1.20 mV (Fig. 2B and C). The embedding of PMVs further increased the size to 153.361 ± 2.83 nm and reduced the potential negativity to − 21.53 ± 0.87 mV (Fig. 2B and C). These changes indicated that LPs achieved RvD1 loading and successful fusion with PMVs. The polydispersity index (PDI) which indexed the homogeneity in the size distribution was also texted in this study, nanoparticles at each stage all have good uniformity (Fig. 2D). The size of PLP-RvD1 was investigated in PBS and PBS containing 20% FBS at room temperature for 7 days (Additional file 1: Fig. S1). We observed that the addition of serum did not change the size of the nanoplatform, suggesting that PLP-RvD1 may be stable in vivo.

The loading efficiency of RvD1 and its release from PLP-RvD1 were detected by HPLC. The results showed that the loading efficiency of RvD1 was about 12.19%, which resulted in a final RvD1 concentration of 2.54 ug/ml (1 mg liposome/ml) for our nano-formulations. In the release assay, we observed a gradual release of RvD1 from PLP-RvD1 in either PBS or 20% serum, reaching 90% at 24 h (Fig. 2E). However, in PBS containing pathological concentration (100 uM) H_2_O_2_, the content of PLP-RvD1 was released explosively, reaching more than 80% within 30 min (Fig. 2E). The non-ROS-responsive control (without diselenide bond) had no burst release effect (Fig. 2E). The above results show that PLP-RvD1 can achieve the responsive release of RvD1 in a high ROS environment, while the addition of 20% serum (simulating the in vivo environment) does not affect the release of its contents. Myocardial ischemia–reperfusion injury is accompanied by a large amount of ROS production [4]. Therefore, compared with traditional drug delivery strategies, the embedding of ROS-responsive components in our delivery platform endows the steric specificity of RvD1 release and alleviates the safety hazards of non-specific drug delivery to a certain extent [34].

The retention of platelet membrane proteins is a prerequisite for PLP-RvD1 to inherit the ability of platelets to interact with macrophages. We used sodium dodecylsulfate polyacrylamide gel electrophoresis (SDS-PAGE) to determined total membrane proteins on PLP-RvD1 first. As shown in Fig. 2F, PLP-RvD1 retained almost all of the platelet membrane proteins compared with PMVs. Then, the retention of key proteins that mediate the interaction of platelets and macrophages (CD42c, P-selectin, integrin GP IIb/IIIa and GP Ibα) in PLP-RvD1 was further confirmed by Western Blot (Fig. 2G) [35]. In addition, to verify the correct orientation of membrane proteins in PLP-RvD1, we used Texas Red-X-conjugated wheat germ agglutinin (WGA) as a probe to detect glycosylation sites in the extracellular segment of membrane proteins. PLTs, PMVs, and PLP-RvD1, but not LP-RvD1 could be stained with WGA (Fig. 2H), indicating that the glycoproteins were expressed on the surface of PLP-RvD1 with the right orientation. These results indicate that PLP-RvD1 retain the same membrane protein composition and right orientation as PMVs, and therefore has the potential to interact with macrophages like PLTs.

### PLP-RvD1 adherence to macrophages in vitro

**Fig. 3:**
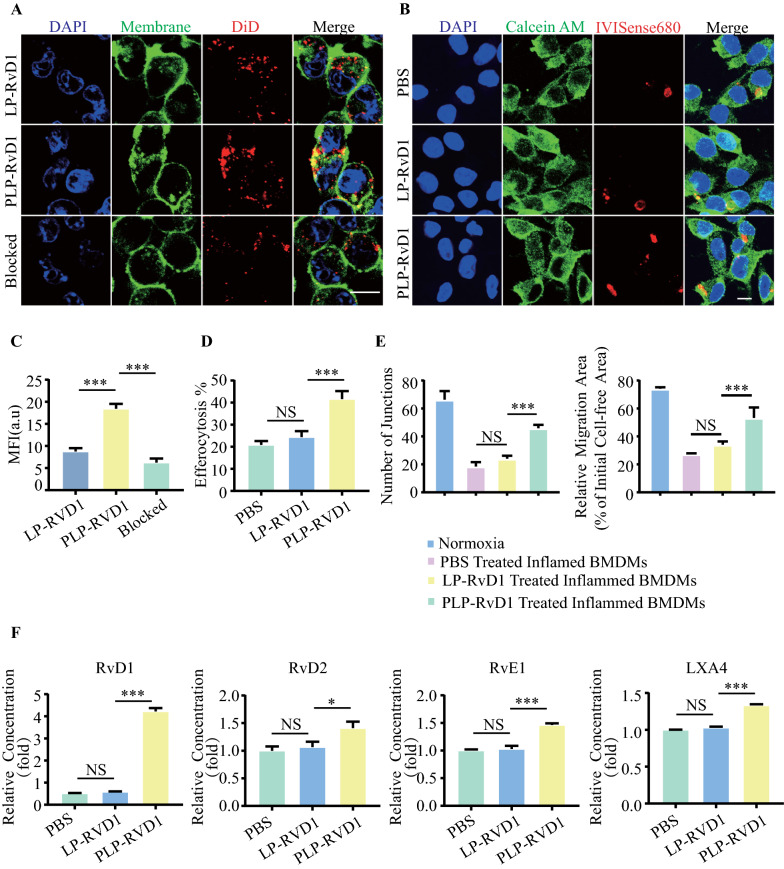
Targeting ability and functional regulation of PLP-RvD1 to BMDMs in vitro. **A** The binding ability of DiD-labeled LP-RvD1, PLP-RvD1 and anti-P-selectin blocked PLP-RvD1 to inflamed BMDMs was detected by fluorescent microscope and **C** statistically analyzed by ImageJ (n = 3 per group). Red, DiD (on liposome); Green, Cell membrane. Scalar bar, 10 µm. **B** The efferocytosis ability of BMDMs to apoptotic cardiomyocytes after PBS, LP-RvD1 or PLP-RvD1 treatment was detected by fluorescence microscopy images and **D** statistically analyzed by ImageJ (n = 3 per group). Green, Calcein AM-labeled BMDMs; Red, IVISense 680-labeled apoptotic cardiomyocytes. Scalar bar, 10 µm. **E** Quantification of capillary formation and cell migration in HUVECs after co-culture with PBS, LP-RvD1 or PLP-RvD1-treated Inflammed BMDMs (n = 3 per group). **F** Determination of related SPMs molecules (RvD1, RvD2, RvE1, LXA4) in culture medium of efferocytosis assay by ELISA (n = 3 per group). Results are presented as mean ± SD. ^NS^P > 0.05, *P < 0.05, **P < 0.01, ***P < 0.001

To verify whether PLP-RvD1 inherits the ability of platelets to interact with macrophages, we used immunofluorescence staining to test the binding ability of PLP-RvD1 to inflamed murine bone marrow-derived macrophages (BMDMs) in vitro. Inflamed BMDMs were incubated with 200 ul DiD (on liposome) labeled LP-RvD1, PLP-RvD1 or anti-P-selectin blocked PLP-RvD1 for 30 min, respectively. As shown in Fig. 3A, LP-RvD1 is rapidly endocytosed by BMDMs, while PLP-RvD1 (anti-P-selectin blocked or not) is mainly anchored on the membrane surface of BMDMs. The DiD mean fluorescence intensity (MFI) of PLP-RvD1-treated cells was 2.22-fold higher than that of LP-RvD1-treated cells (Fig. 3C), whereas anti-P-selectin blockade significantly reduced PLP-RvD1 binding to BMDMs (Fig. 3C), suggesting that PLP-RvD1 inherits the ability of platelets to interact with macrophages.

### PLP-RvD1 promotes efferocytosis of apoptotic cardiomyocytes by BMDMs in vitro

To verify whether PLP-RvD1 could release RvD1 responsively in a high ROS environment, thereby affecting the efferocytosis ability of BMDMs, we pretreated BMDMs with 200 ul PBS, LP-RvD1 or PLP-RvD1 for 30 min, and then administered a pathological dose (100 uM) of H2O2 to induce responsive release of RvD1 followed by 6 h incubation. At the same time, IVISense 680 (red)-labeled mouse cardiomyocyte HL-1 was induced to apoptosis by staurosporine. Then IVISense 680 (red)-labeled apoptotic cardiomyocytes were incubated with Calcein AM (green)-labeled BMDMs, and the efferocytosis index, the percentage of cardiomyocyte-associated macrophages, was calculated according to a previously reported method to evaluate the efferocytosis ability[32]. The results showed that only the PLP-RvD1 group could significantly enhance the efferocytosis ability of BMDMs, but LP-RvD1 could not (Fig. 3B and D). This may be attributed to the fact that LP-RvD1 is rapidly phagocytosed by BMDMs and RvD1 cannot be released into the medium in response to ROS, and then acts on its corresponding receptors on the surface of BMDMs.

### PLP-RvD1 induces a pro-angiogenic phenotype in BMDMs in vitro

In this study, we introduced a transwell system consisting of pretreated BMDMs as mentioned in the previous section in the upper chamber and HUVECs after hypoxia stimulation in the lower layer to explore the effect of PLP-RvD1-treated macrophages on angiogenesis in vitro. After 12 h of coculture, the HUVECs were collected and conducted to tube formation and migration test. PLP-RVD1-treated BMDMs significantly stimulated capillary formation in HUVECs cultured under hypoxic conditions, whereas LP-RvD1 showed no significant difference compared to the PBS group (Additional file 1: Fig. 2A, Fig. 3E). Meanwhile, the migration assay also obtained similar results. PLP-RvD1-treated BMDMs significantly enhanced endothelial cell migration ability, while LP-RvD1-treated BMDMs did not (Additional file 1: Fig. 2B, Fig. 3E). HUVECs cultured under normoxia were adopted as positive control. These data suggest that PLP-RvD1 promotes the transformation of macrophages into a pro-angiogenic phenotype.

### PLP-RvD1 promotes production of SPMs in vitro

Both RvD1 and efferocytosis have been reported to promote the production of SPMs [13, 14, 36]. We detected the concentration of SPMs (RvD1, RvD2, RvE1 and LXA4) in culture medium of efferocytosis assay by ELISA. We observed that PLP-RvD1 treatment could significantly upregulate all 4 target molecules, whereas LP-RvD1 could not (Fig. 3F). Notably, the upregulation of RvD1 was much higher than that of other SPMs, which may be partly supplemented by the responsive release of RvD1 in PLP-RvD1 bound on BMDMs.

### Biodistribution and targeting specificity of PLP-RvD1 in vivo

**Fig. 4:**
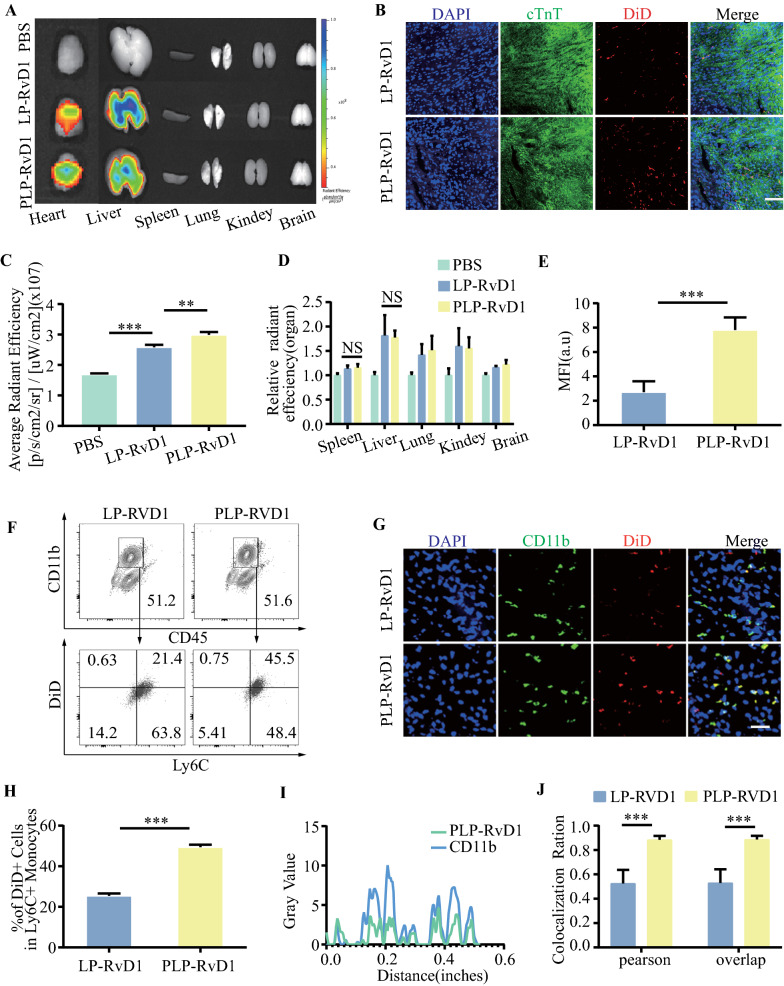
Biodistribution and targeting specificity of PLP-RvD1 in vivo. **A** IVIS images of LP-RvD1 or PLP-RvD1 accumulated in the main organs after i.v.injected in MI/R mice. **B** CLSM images of heart sections showing the accumulation of LP-RvD1 and PLP-RvD1 in injured heart after immuno-stained with cardiac troponin T (cTnT). Red, DiD (on liposome) labeled nanoparticles; Green, cTnT. Scalar bar, 50 µm. **C** Quantitative analysis of the accumulation of LP-RvD1 or PLP-RvD1 in heart and **D** other major organs of MI/R induced mice based on images in (A) (n = 6 per group). **E** Statistical analysis of fluorescence images in (B) by ImageJ (n = 6 per group). **F** Flow cytometry analysis of the binding ability of PLP-RvD1 to Ly6C + monocytes in the blood circulation after intravenous injection at 72 h post-MI/R and (H) was further quantified (n = 6 per group). **G** CLSM images of heart sections showing the colocalization between monocytes/macrophages and LP-RvD1 or PLP-RvD1 after immuno-stained with CD11b. Red, DiD (on liposome) labeled nanoparticles; Green, CD11b. Scalar bar, 20 µm. **I** The plot profile, **J** pearson’s correlation and overlap coefficient were quantified after colocalization analysis of images from **G** via Image J (n = 6 per group). Results are presented as mean ± SD. ^NS^*P* > 0.05, *P < 0.05, **P < 0.01, ***P < 0.001.

To systematically evaluate the in vivo performance of PLP-RvD1, the blood circulation profile was investigated first. 200 ul DiD labeled LP-RvD1 or PLP-RvD1 were injected into healthy mice through tail vein injection. As shown in Additional file 1: Fig. S3, the presence of platelet membrane components extends the biological half-life of PLP-RvD1. The distribution of DiD (on liposome) labeled LP-RvD1 or PLP-RvD1 in the major organs of MI/R induced mice was examined by IVIS (Fig. 4A). The results showed that the accumulation of PLP-RvD1 in the injured heart was much higher than all other groups (Fig. 4C). There was no significant difference in their distribution in other major organs (Fig. 4D). Immunofluorescence staining further confirmed the better accumulation of PLP-RvD1 in the heart injury area, and it was mainly distributed in the infarct border area (Fig. 4B, E).

To further explore the underlying mechanism of the selective accumulation of PLP-RvD1 in the injured heart, we used flow cytometry to verify the ability of DiD (on liposome) labeled PLP-RvD1 to Ly6C + monocytes in mice blood circulation after tail vein injection at 72 h post-MI/R (Fig. 4F). The proportion of DiD + Ly6C + cells in PLP-RvD1 group was significantly higher than that of LP-RvD1 group (Fig. 4H), indicating that the presence of platelet membrane components effectively enhanced the binding of PLP-RvD1 with Ly6C + monocytes. To verify whether PLP-RvD1 can reach the heart injury area through carrying of monocytes, we used immunofluorescence staining to analyze the co-localization of DiD (on liposomes) labeled LP-RvD1 or PLP-RvD1 with monocytes/macrophages in injured hearts. The results indicated that compared with LP-RvD1, PLP-RvD1 showed more pronounced co-localization with monocytes/macrophages in the injured heart (Fig. 4G, I, J).

Collectively, these results suggest that PLP-RvD1 can utilize monocyte pathophysiological chemotaxis to actively target injured hearts by hijacking it in the blood circulation. Compared with EPR effect-dependent drug delivery methods, our in vivo hijacking strategy is more suitable for the time window when the number of macrophages reaches the peak during natural inflammation, which is helpful for the curative effect [27]. Compared to cell therapy with a backpack, our hijacking strategy is more cost-effective and safer [37].

### PLP-RvD1 promotes efferocytosis, angiogenesis and SPM production in vivo

**Fig. 5:**
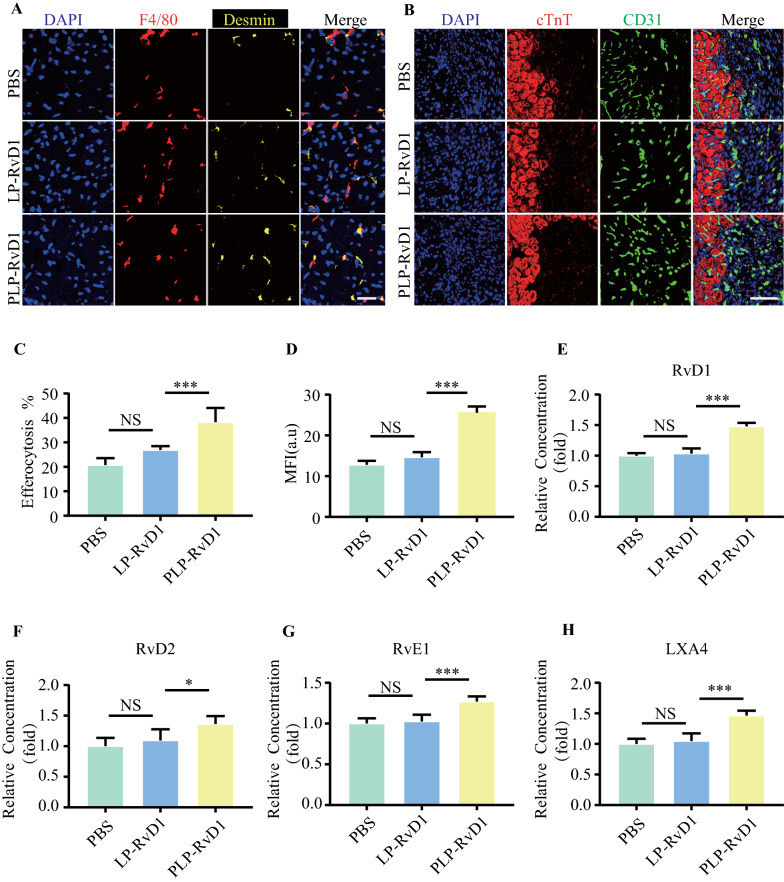
PLP-RvD1 promotes efferocytosis, angiogenesis and the synthesis of SPMs in vivo. **A** The efferocytosis ability of macrophages to apoptotic cardiomyocytes in vivo after PBS, LP-RvD1 or PLP-RvD1 treatment was detected by fluorescence microscopy images and **C** the efferocytosis index was calculated. (n = 6 per group). Red, F4/80; yellow, Desmin, a cardiomyocyte marker. Scalar bar, 20 µm. **B** CLSM images of CD31 + signals in MI/R injured murine heart sections after PBS, LP-RvD1, or PLP-RvD1 administered and **D** statistically analyzed by ImageJ (n = 6 per group). Red, cTnT; Green, CD31. Scalar bar, 50 µm. **E**–**H** Determination of related SPMs molecules (RvD1, RvD2, RvE1, LXA4) in cardiac tissue homogenate by ELISA after treated with PBS, LP-RvD1 or PLP-RvD1, respectively (n = 6 per group). Results are presented as mean ± SD. ^NS^P > 0.05, *P < 0.05, **P < 0.01, ***P < 0.001

We conducted a series of validations on the in vivo efficacy of PLP-RvD1. Firstly, we employed a previously reported experimental approach to evaluate the effect of PLP-RvD1 on macrophage efferocytosis in MI/R mouse hearts [32]. Compared with the PBS group, the efferocytosis index was significantly increased in the PLP-RvD1 group, while there was no significant difference in the LP-RvD1 group (Fig. 5A, C). Secondly, the assessment of angiogenesis in the injured heart also yielded similar results. The PLP-RvD1 group possess the most microvascular density (MFI of CD31 + area), suggesting the best pro-angiogenesis effect (Fig. 5B, D). Unsurprisingly, the production of SPMs in the cardiac homogenate of the PLP-RvD1 group was significantly higher than that of the PBS and LP-RvD1 groups, with no statistical difference between the latter two groups (Fig. 5E–H).

### Cardiac protection efficiency of PLP-RvD1

**Fig. 6:**
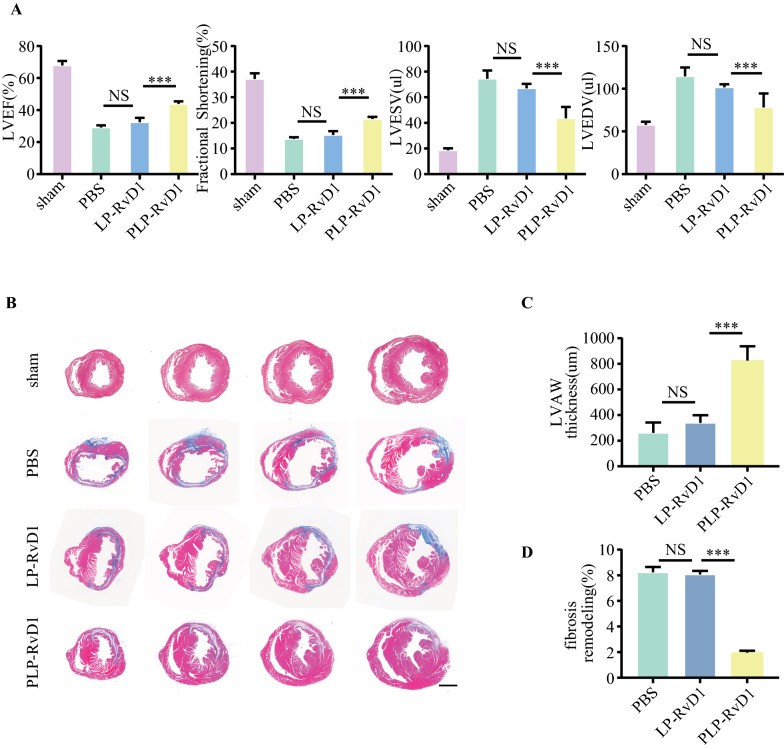
Cardiac protection efficiency of PLP-RvD1. **A** Cardiac function was assessed by echocardiography at 4 weeks after treatment (n = 6 per group). **B** Masson staining of MI/R heart paraffin sections at 4 weeks after various treatments. Scalar bar, 1 mm. **C** LVAW thickness and **D** fibrosis remodeling in (**B**) was quantified by using ImageJ software (n = 6 per group). Results are presented as mean ± SD. ^NS^P > 0.05, *P < 0.05, **P < 0.01, ***P < 0.001

To evaluate the therapeutic benefits of PLP-RvD1, cardiac function of MI/R induced mice was detected by echocardiography after 4 weeks of PBS, LP-RvD1 or PLP-RvD1 administered. As shown in Fig. 6A, the LVEF of the PLP-RVD1 group was preserved the most when compared with other groups (43.85 ± 1.57% vs 32.34 ± 2.84% vs 29.09 ± 1.32%, PLP-RvD1 vs LP-RvD1 vs Ctrl), while the LVFS increased by 6.19% and 8.07% vs LP-RvD1 and control group, respectively (21.47 ± 0.89% vs 15.28 ± 1.50% vs 13.67 ± 0.71%, PLP-RvD1 vs LP-RvD1 vs Ctrl). Other echocardiographic parameters including left ventricular end-systolic volume (LVESV) and left ventricular end-diastolic volume (LVEDV) showed similar results (Fig. 6A). Then pathological remodeling was also evaluated by Masson staining. The preserved left ventricular anterior wall (LVAW) thickness and fibrosis remodeling in different layers of heart paraffin sections were quantified (Fig. 6B). The cardiac remodeling was improved greatest in PLP-RvD1 group, which had a thicker infarcted wall and smaller scar size than any other groups (Fig. 6B–D). These results indicated that PLP-RvD1 could effectively improve ventricular remodeling in MI/R mice.

### Biosafety assessment of PLP-RvD1

**Fig. 7:**
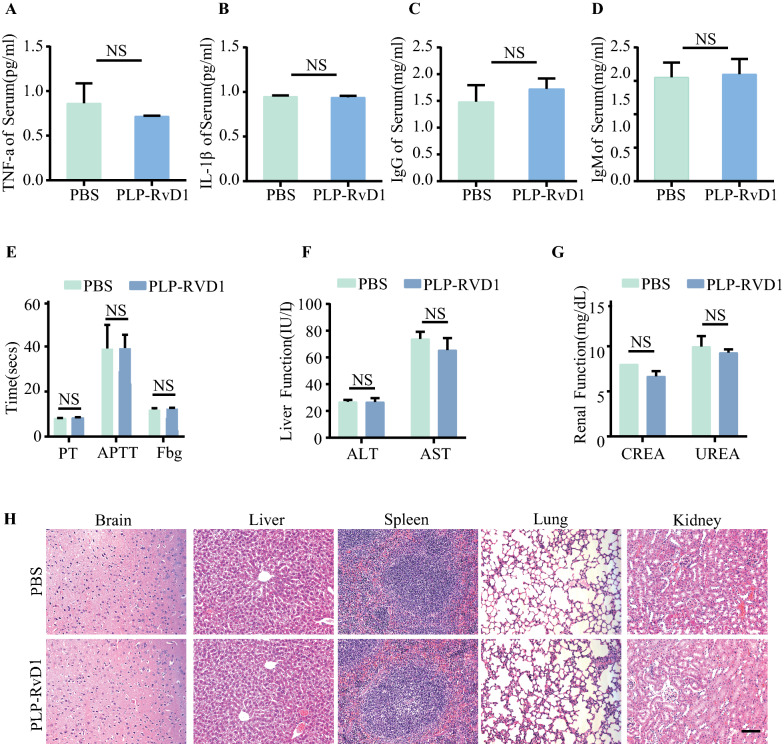
Biosafety verification of PLP-RvD1. **A**, **B** Serum concentration of inflammatory cytokines (IL-1β, TNF-α) of healthy mice detected by ELISA assay at 3 d post administration of PBS or PLP-RvD1 (n = 6 per group). **C**, **D** ELISA assay of immune response indicated by serum level of general IgG and IgM of healthy mice with or without PLP-RvD1 treatment (n = 6 per group). **E** Evaluation of the effect of PLP-RvD1 on clotting function (n = 6 per group). Biochemical test of **F** liver and **G** renal function of healthy mice after PBS or PLP-RVD1 administered (n = 6 per group). **H** Histology characteristics of major organs of PBS or PLP-RvD1 treated healthy mice were detected by HE staining. Scalar bar, 100 μm. Results are presented as mean ± SD. ^NS^*P* > 0.05

To verify the biosafety of PLP-RvD1, we tested a series of safety parameters in healthy mice after treatment with 200 ul PBS or PLP-RvD1. To explore whether PLP-RvD1 treatment induces acute inflammatory responses, we quantified the serum levels of TNF-α, IL-1 by ELISA assay at 3 days, and no significance was observed among these groups (Fig. 7A, B). Then, the general antibodies, namely, IgG and IgM were also measured by ELISA assay, and the results suggested no potential immune response after PLP-RvD1 administration (Fig. 7C, D). In addition, coagulation-related parameters, including levels of APTT, PT, and Fbg, showed no significant differences between groups (Fig. 7E). With respect to organ toxicity, biochemistry analysis showed that ALT, AST, CREA, and UREA demonstrated no difference between PLP-RvD1 and PBS group, indicating the hepatic and kidney functions were not affected by PLP-RvD1 treatment (Fig. 7F, G). Meanwhile, compared with PBS group, no histopathological changes were observed in major organs of heathy mice treated with PLP-RvD1 (Fig. 7H). These results suggest that PLP-RvD1 has no potential organ toxicity. Accordingly, the biosafety of PLP-RvD1 was up to standard, which is essential for its potential clinical application.

## Conclusion

We developed an active targeting system consisting of platelet membrane chimeric ROS-responsive liposomes that integrates the targeted delivery and responsive release of RvD1. After intravenous injection, PLP-RvD1 can reach the site of cardiac injury by riding circulating monocytes in chemotaxis, and release RvD1 explosively under the stimulation of local high ROS levels, thereby promoting efferocytosis, angiogenesis and SPMs production. In vivo application of PLP-RvD1 can effectively improve ventricular remodeling and preserve cardiac function in MI/R induced mice. Our formulation integrates drug biological stability, targeted delivery and controlled release, which endow it with great clinical translational value.

## Supplementary Information

**Additional file 1: Figure S1. **Stability of nanovesicles in PBS and PBS with 20% of fetal bovine serum (FBS). Nanovesicle sizes were measured using dynamic light scattering (n=3 per group). Results are presented as mean ± SD. **Figure S2. **The promotion of PLP-RvD1 treated macrophages to angiogenesis. (**A**) Capillary tube formation and (**B**) cell migration of HUVECs after cocultured with PBS, LP-RvD1 or PLP-RvD1 treated BMDMs. HUVECs cultured under normoxia were set as controls. Scalar bar, 100 μm and 200 μm, respectively. **Figure S3. **Circulation profiles of LP-RvD1 and PLP-RvD1 in healthy mice after intravenous injection (n=6 per group). Results are presented as mean ± SD.

## Data Availability

The data that support the findings of this study are available from the corresponding authors upon reasonable request.
