# Supplementary material for: Targeted delivery and ROS-responsive release of Resolvin D1 by platelet chimeric liposome ameliorates myocardial ischemia–reperfusion injury
Source: J Nanobiotechnology. 2022 Oct 20;20:454. doi: 10.1186/s12951-022-01652-x (PMC9585729; doi:10.1186/s12951-022-01652-x)
Supplement: Supplementary file 1 — Additional file 1: Figure S1. Stability of nanovesicles in PBS and PBS with 20% of fetal bovine serum (FBS). Nanovesicle sizes were measured using dynamic light scattering (n=3 per group). Results are presented as mean ± SD. Figure S2. The promotion of PLP-RvD1 treated macrophages to angiogenesis. (A) Capillary tube formation and (B) cell migration of HUVECs after cocultured with PBS, LP-RvD1 or PLP-RvD1 treated BMDMs. HUVECs cultured under normoxia were set as controls. Scalar bar, 100 μm and 200 μm, respectively. Figure S3. Circulation profiles of LP-RvD1 and PLP-RvD1 in healthy mice after intravenous injection (n=6 per group). Results are presented as mean ± SD. [file 12951_2022_1652_MOESM1_ESM.docx]

**Targeted delivery and ROS-responsive release of Resolvin D1 by platelet chimeric liposome ameliorates myocardial ischemia-reperfusion injury**

Xueyi Weng ^1,2#^, Haipeng Tan ^1,2#^, Zheyong Huang ^1,2#^, Jing Chen ^1,2^, Ning Zhang ^1,2^, Qiaozi Wang^1,2^, Qiyu Li ^1,2^, Jinfeng Gao ^1,2^, Dili Sun ^1,2^, Wusiman Yakufu ^1,2^, Zhengmin Wang ^1,2^, Weiyan Li ^1,2^, Guangrui Zhu^1,2^, Zhiqing Pang ^3*^, Yanan Song ^1,2*^, Juying Qian ^1,2*^, and Junbo Ge ^1,2,4^

1 Department of Cardiology, Zhongshan Hospital, Fudan University, Shanghai Institute of Cardiovascular Diseases, 180 Feng Lin Road, Shanghai 200032, China

2 National Clinical Research Center for Interventional Medicine& Shanghai Clinical Research Center for Interventional Medicine, 180 Feng Lin Road, Shanghai 200032, China

3 School of Pharmacy, Fudan University, Key Laboratory of Smart Drug Delivery, Ministry of Education, 826 Zhangheng Road, Shanghai 201203, China

4 Institute of Biomedical Science, Fudan University, 180 Feng Lin Road, Shanghai 200032, China

#These authors contributed equally to this work


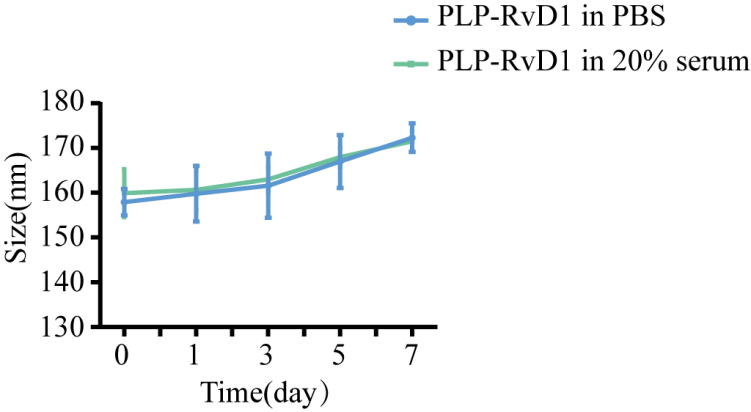


**Figure S1** Stability of nanovesicles in PBS and PBS with 20% of fetal bovine serum (FBS). Nanovesicle sizes were measured using dynamic light scattering (n=3 per group). Results are presented as mean ± SD.


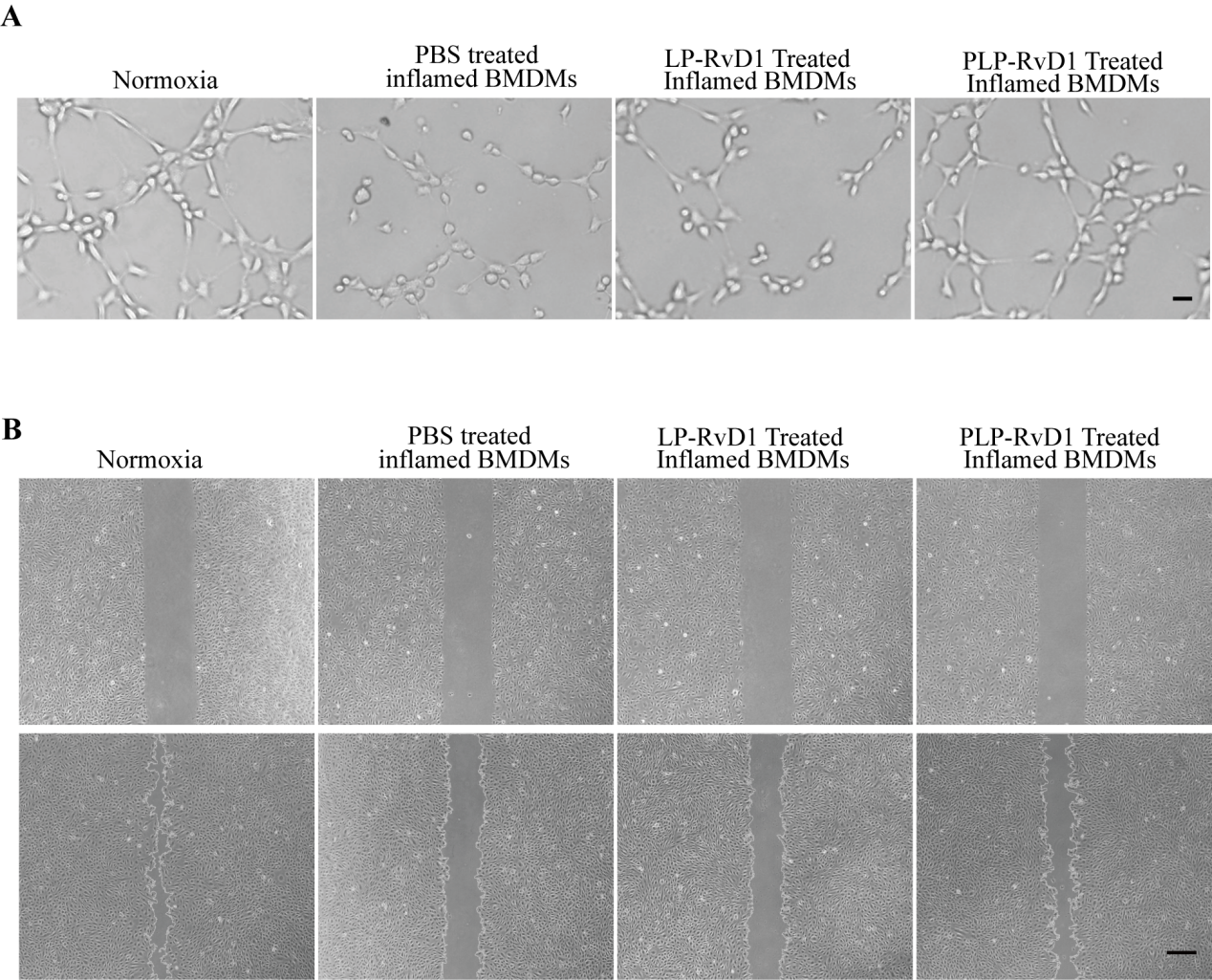


**Figure S2** The promotion of PLP-RvD1 treated macrophages to angiogenesis. (**A**) Capillary tube formation and (**B**) cell migration of HUVECs after cocultured with PBS, LP-RvD1 or PLP-RvD1 treated BMDMs. HUVECs cultured under normoxia were set as controls. Scalar bar, 100 μm and 200 μm, respectively.


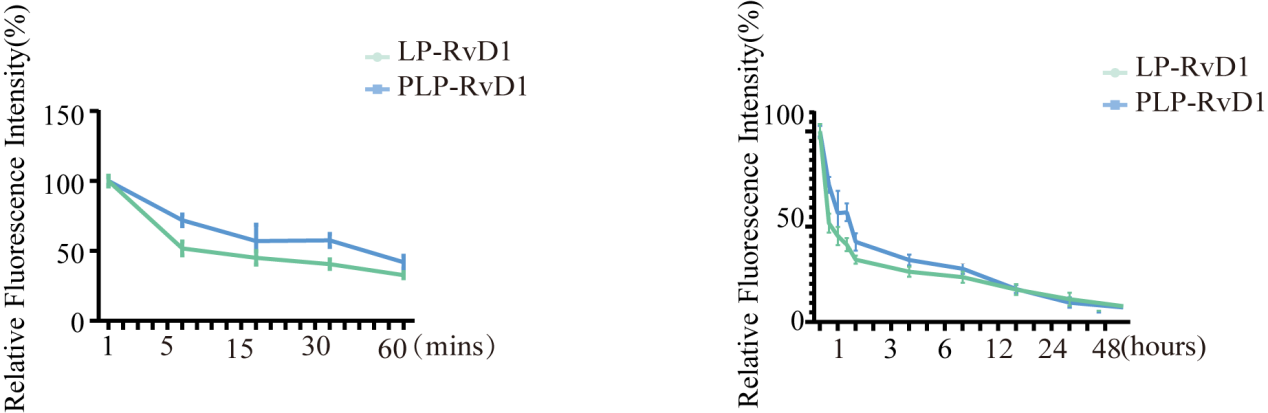


**Figure S3** Circulation profiles of LP-RvD1 and PLP-RvD1 in healthy mice after intravenous injection (n=6 per group). Results are presented as mean ± SD.
